# Key Genes *FECH* and *ALAS2* under Acute High-Altitude Exposure: A Gene Expression and Network Analysis Based on Expression Profile Data

**DOI:** 10.3390/genes15081075

**Published:** 2024-08-14

**Authors:** Yifan Zhao, Lingling Zhu, Dawei Shi, Jiayue Gao, Ming Fan

**Affiliations:** 1 School of Information Science and Engineering, Lanzhou University, Lanzhou 730000, China; zhaoyifan11163@163.com; 2 Beijing Institute of Basic Medical Sciences, Beijing 100850, China; linglingzhuamms@126.com; 3 School of Automation, Beijing Institute of Technology, Beijing 100850, China; daweishi@bit.edu.cn

**Keywords:** high altitude, acclimatization, gene expression, mRNA expression profile, biomarkers

## Abstract

High-altitude acclimatization refers to the physiological adjustments and adaptation processes by which the human body gradually adapts to the hypoxic conditions of high altitudes after entering such environments. This study analyzed three mRNA expression profile datasets from the GEO database, focusing on 93 healthy residents from low altitudes (≤1400 m). Peripheral blood samples were collected for analysis on the third day after these individuals rapidly ascended to higher altitudes (3000–5300 m). The analysis identified significant differential expression in 382 genes, with 361 genes upregulated and 21 downregulated. Further, gene ontology (GO) annotation analysis and Kyoto Encyclopedia of Genes and Genomes (KEGG) pathway enrichment analysis indicated that the top-ranked enriched pathways are upregulated, involving blood gas transport, erythrocyte development and differentiation, and heme biosynthetic process. Network analysis highlighted ten key genes, namely, *SLC4A1*, *FECH*, *EPB42*, *SNCA*, *GATA1*, *KLF1*, *GYPB*, *ALAS2*, *DMTN*, and *GYPA*. Analysis revealed that two of these key genes, *FECH* and *ALAS2*, play a critical role in the heme biosynthetic process, which is pivotal in the development and maturation of red blood cells. These findings provide new insights into the key gene mechanisms of high-altitude acclimatization and identify potential biomarkers and targets for personalized acclimatization strategies.

## 1. Introduction

High-altitude regions possess a uniquely complex ecological environment. As altitude increases, atmospheric pressure exponentially decreases, and oxygen levels in the air diminish accordingly. Globally, for those traveling to high-altitude areas for work or tourism, acute mountain sickness (AMS) triggered by hypoxic exposure poses a significant health challenge. The incidence of AMS can reach 50–80% [1], influenced by a multitude of factors such as individual variability, altitude, duration of exposure, and pre-acclimatization practices [2], with rapid ascent to high altitudes being a critical risk factor [3]. At the systemic level, acute hypoxic exposure triggers various physiological responses to aid the body’s adaptation to hypoxia. Under low pressure and hypoxia, peripheral sensors enhance their stimulatory effects on the respiratory center, leading to compensatory ventilation responses such as increased depth and rate of breathing. In response to hypoxia at high altitudes, the body’s adrenergic system plays a compensatory role by enhancing sympathetic nerve activity. This leads to increased stimulation of adrenergic receptors, which raises heart rate and boosts cardiac output, thereby maintaining oxygen delivery despite lower ambient oxygen levels [4]. Blood flow redistribution occurs; the vascular response to hypoxia varies by location, with hypoxia causing pulmonary vasoconstriction and peripheral circulation vasodilation [5], where pulmonary vasoconstriction is a primary factor in the onset and progression of pulmonary hypertension [6]. Acute hypoxia stimulates the sympathetic nervous system, causing vasoconstriction in the skin, liver, and spleen vessels, leading to more blood entering the systemic circulation. This results in an increase in the total amount of red blood cells and hemoglobin participating in the circulation [7,8].

Rapid ascent in altitude leads to acute hypoxia in tissues and organs. At the cellular level, hypoxia activates the Hypoxia-Inducible Factor (HIF) pathway. Under normoxic conditions, prolyl hydroxylase modifies HIF-1α post-translationally, and the hydroxylated HIF-1α is susceptible to proteasomal degradation. In hypoxic conditions, this hydroxylation is inhibited, resulting in the accumulation of HIF-1α within the cell [9]. HIF-1α forms a dimer with HIF-1β and then translocates into the nucleus, where HIF-1 stimulates the expression of hundreds of genes that facilitate both autonomous and non-autonomous cellular adaptation to hypoxia, thereby supporting the maintenance of tissue oxygen supply. For instance, in high-altitude regions, the stabilization of HIF-1α leads to upregulated transcription of erythropoietin (EPO) and vascular endothelial growth factor (VEGF) [10], promoting erythropoiesis and angiogenesis [11,12]. HIF-1 is also involved in regulating glucose metabolism under hypoxic conditions. It upregulates glucose transport proteins such as GLUT-1, enhancing glucose uptake and utilization, and activates hexokinases 1 and 2 (HK1 and HK2), pyruvate kinase (PKM), and lactate dehydrogenase (LDHA), thereby promoting glycolysis. Because of the lack of oxygen, the tricarboxylic acid cycle and the electron transport chain are also inhibited [13].

Despite existing research into the mechanisms of human acclimatization to high altitudes, the key genes involved, and their interactions remain incompletely elucidated. From a molecular perspective, rapid acclimatization to high-altitude environments involves the activation of multiple genes and signaling pathways [14,15]; these genes and pathways form an interactive network that collectively responds to hypoxic conditions [16,17].

This study utilized three datasets from the Gene Expression Omnibus (GEO) database to systematically investigate gene expression changes following a rapid ascent from low to high altitudes, thereby revealing key genes involved in the human acclimatization process to high-altitude environments. Through data integration and analysis of molecular interactions, pathways that were significantly enriched were identified, including those involved in blood gas transport such as carbon dioxide transport, the heme biosynthetic process, and the hemoglobin complex. Pathways involved in erythrocyte development and differentiation were also highlighted, such as erythrocyte development, erythrocyte differentiation, and the spectrin-associated cytoskeleton. Additionally, pathways related to energy and metabolism, including metabolic pathways and porphyrin metabolism, were identified. From these pathways, the following ten key genes were selected: SLC4A1, FECH, EPB42, SNCA, GATA1, KLF1, GYPB, ALAS2, DMTN, and GYPA. This research brings attention to genes and their synergistic interactions that have not been sufficiently discussed in the existing studies.

## 2. Materials and Methods

### 2.1. Data Collection and Preliminary Processing

**Figure 1:**
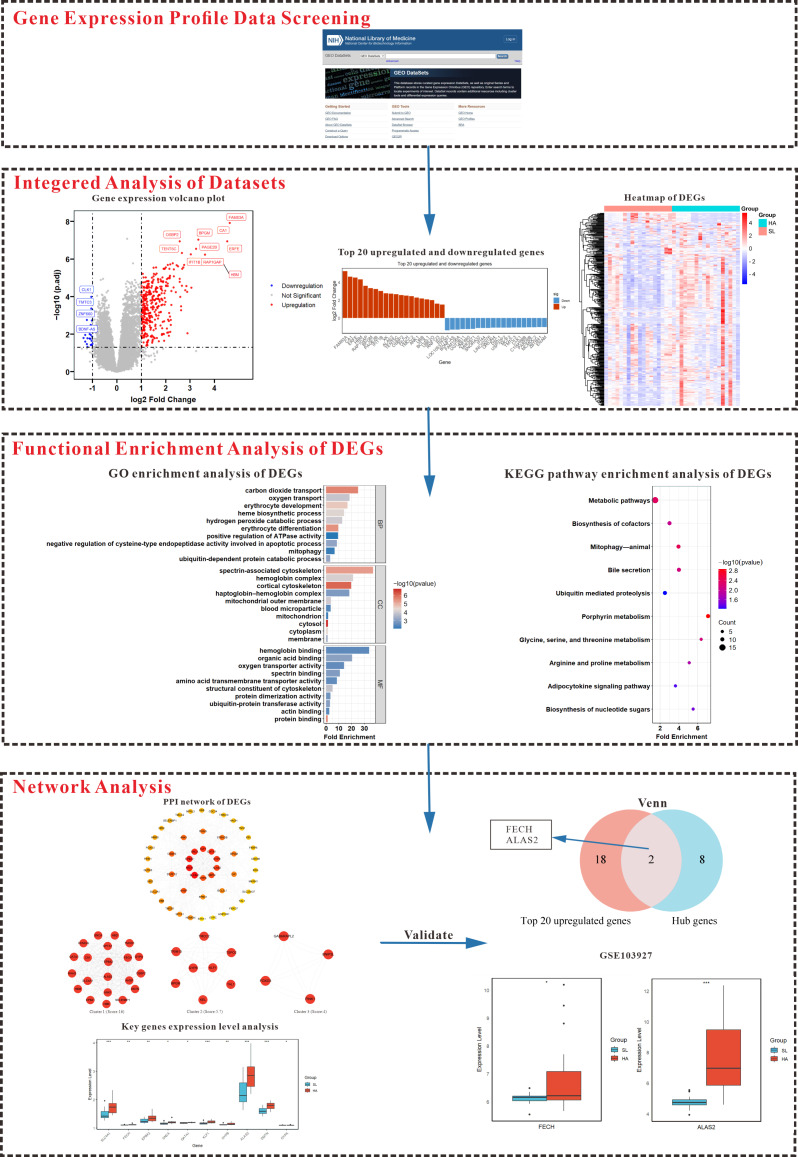
Summary of the overall workflow and related results of the gene expression analysis. This figure illustrates a comprehensive workflow starting with data selection from the GEO database, followed by visualization of DEGs. It progresses to functional enrichment analyses using GO and KEGG, identifying critical pathways like carbon dioxide transport and the heme biosynthetic process. Subsequent network analysis identifies three highly interconnected sub-networks and ten key genes, with their expression levels depicted in box plots. The Venn diagram highlights the overlap between the top 20 upregulated genes and the hub genes. Additionally, the expression of two pivotal genes, *FECH* and *ALAS2*, was validated using the GSE103927 dataset, showing significant upregulation and underscoring their roles in high-altitude acclimatization. *** denotes very significant (*p* < 0.001), ** denotes significant (*p* < 0.01), and * denotes moderately significant (*p* < 0.05).

Using the keywords “gene expression” and “high altitude” in the GEO database, three mRNA expression profile datasets that met the criteria and were on the same platform (GPL11154) were selected from the GEO database in NCBI. By integrating these datasets to remove batch effects (Figure S1), a consolidated dataset was obtained for further analysis. The datasets used in this study (GSE133702, GSE103940, GSE75665) were obtained from the GEO database, as part of prior research that received ethical approval from designated committees. Although specific approval numbers are not disclosed, the original publications confirm adherence to ethical guidelines. The study population consisted entirely of healthy male residents from low altitudes (≤1400 m) who had never traveled to high-altitude regions and had no known history of cardiovascular or other diseases. Physiological data and peripheral blood samples (SL group) were initially collected at low altitudes. On the third day after rapidly ascending to high altitudes (3000 m–5300 m), peripheral blood samples were again collected (HA group). The basic characteristics of all participants are shown in Table 1. The entire analysis process of this study was shown in Figure 1.

### 2.2. Collection and Preliminary Processing of External Datasets

The GSE103927 gene expression profile dataset was collected from the GEO database. Participants were carefully selected based on the following strict exclusion criteria: being born at altitudes over 1500 m; recent travel (within the last three months) above 1000 m; current use of prescription medications; smoking habits; pregnancy or lactation; history of severe head injuries or migraines; hematologic disorders like sickle cell trait; cardiovascular abnormalities such as cardiac arrhythmia; pulmonary function or diffusion capacity for carbon monoxide below 90% of predicted; or failure to meet the age and gender standards of the Army Physical Fitness Test (APFT). Similar to the datasets discussed earlier, the GSE103927 dataset adheres to ethical standards previously established by the initial researchers. This study commenced with the collection of physiological data and peripheral blood samples at low altitudes (SL group). We then selected participants for a subsequent collection of peripheral blood samples on the seventh day after a rapid ascent to 5260 m altitude (HA group). This study involved 21 participants (12 males and 9 females, average age of 20.8 years, ranging from 19 to 23 years). Subsequently, key gene expression levels were analyzed using R, and boxplots were created.

### 2.3. Identification of Differentially Expressed Genes

To explore how genes respond to changes in altitude, we conducted an analysis to identify genes that significantly alter their expression levels between low- and high-altitude conditions. This process, known as Differentially Expressed Gene (DEG) analysis, began by examining the gene expression data across our datasets. We employed the Benjamini–Hochberg method to adjust the *p*-values from this analysis, which helped control the false discovery rate. This adjustment was essential to ensure that the genes identified as differentially expressed were indeed likely to be true findings rather than false positives due to random variations in the data. Significant DEGs were defined by a threshold of |Log_2_(Fold Change)| ≥ 1, with an adjusted *p*-value (p.adj) < 0.05. To visually represent and interpret these changes, we generated a volcano plot using the “dplyr” and “ggplot2” packages in R. This plot visually contrasts the magnitude of gene expression changes against their statistical significance, providing a clear and intuitive visualization of the most dramatically altered genes. Additionally, we created a heatmap using the “pheatmap” package to display these DEGs.

### 2.4. Functional Annotation and Enrichment Analysis

DAVID Bioinformatics Resources (https://david.ncifcrf.gov/, accessed on 11 February 2024) was used in enrichment analysis including Gene Ontology (GO) annotation analysis and Kyoto Encyclopedia of Genes and Genomes (KEGG) annotation analysis. The GO analysis was employed in three classes of biological processes (BPs), molecular functions (MFs), and cellular components (CCs). Concurrently, the KEGG analysis connected genes with specific biochemical pathways, illuminating their roles in functional and metabolic activities. The purpose of this functional enrichment analysis was to identify key biological processes and metabolic pathways significantly associated with genes that alter their expression in response to altitude changes. We determined the significance of these associations using p.adj to ensure that the findings were statistically robust and not merely due to random variations.

To process and visualize the data, we employed several R packages, including “readxl” for reading data, “ggplot2” for generating informative bar and bubble plots, and “dplyr” for data manipulation. These tools helped us create visual representations that clearly and effectively illustrate the enrichment levels and the significance of the identified GO terms and KEGG pathways, thus aiding in the interpretation of our results.

### 2.5. Construction and Validation of Protein–Protein Interaction Network

We further constructed a protein–protein interaction (PPI) network using the STRING database, which integrates both known and predicted protein–protein interaction information, encompassing direct (physical) and indirect (functional) connections among proteins. This database was instrumental in exploring potential interactions among the differentially expressed genes identified in our study. For an in-depth analysis of the network, we utilized Cytoscape software, a widely used tool for network visualization and analysis. In Cytoscape, we sorted network nodes by degree centrality of proteins to reflect their importance within the network. Additionally, we employed the MCODE algorithm in Cytoscape to detect densely interconnected regions or subnetworks within the overall network. These subnetworks often represent groups of proteins that collaborate in specific biological functions or pathways. We analyzed gene expression levels related to key proteins in the network by creating boxplots using the “dplyr” and “ggpubr” packages in R, which facilitated the detailed graphical representation of the data. We also generated Venn diagrams to illustrate the overlap elements among our datasets using the web-based application available at http://www.bioinformatics.com.cn/plot_basic_proportional_2_or_3_venn_diagram_028 (accessed on 13 March 2024).

## 3. Results

### 3.1. Identification of Differentially Expressed mRNAs

**Figure 2:**
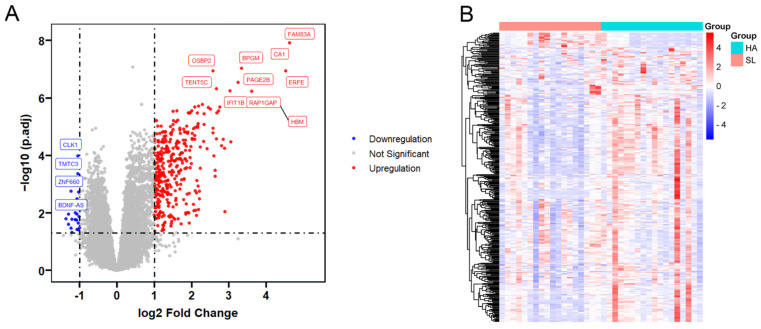
Differentially expressed genes in high-altitude conditions. Each point represents a gene, with the *x*-axis showing the log_2_ fold change (log_2_(FC)) to indicate the magnitude of gene expression changes and the *y*-axis showing the negative log_10_ transformation of the adjusted *p*-value (−log_10_(p.adj)), emphasizing the statistical significance of these changes. (**A**). Significant upregulation and downregulation thresholds are set at |log_2_(FC)| ≥ 1 and p.adj < 0.05. A heatmap illustrates the patterns of gene expression across different samples, with the color scale representing the intensity of gene expression (**B**).

In this study, the integrated dataset was analyzed using the limma package in R to identify and analyze differentially expressed genes in the dataset. During the acclimatization process to high altitudes, 382 DEGs were identified, including 361 upregulated genes and 21 downregulated genes (Figure 2A). This result indicates that a substantial number of genes increased their expression levels. Figure 2B displays the expression levels of DEGs in the dataset.

### 3.2. GO and KEGG Enrichment Analysis

**Figure 3:**
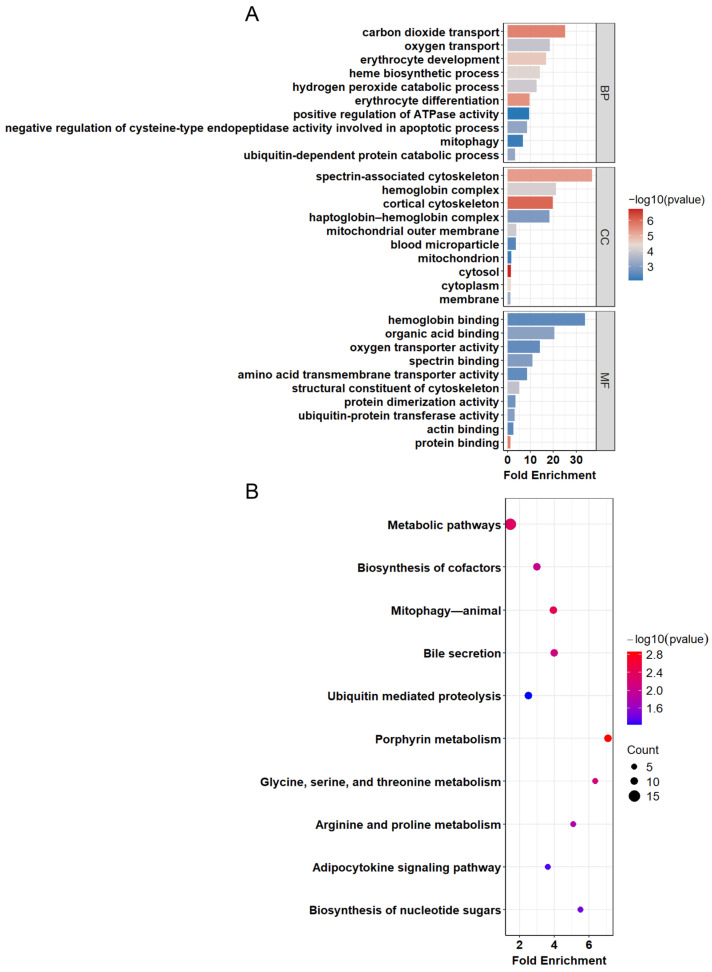
Functional enrichment results of the gene expression data. The GO enrichment results for significant gene expression data under high-altitude conditions are divided into three main categories as follows: BP, CC, and MF. Each bar graph represents the enrichment level of a specific GO term, with the length of the bar indicating the fold enrichment, which represents the enrichment ratio compared to the expected random value. The depth of the color represents the logarithmic value of the *p*-value (**A**). The KEGG analysis results for significantly differentially expressed genes, displaying the fold enrichment of various KEGG pathways and their corresponding statistical significance (*p*-value), with the size of the dots representing the number of genes enriched in each pathway (**B**).

To gain a deeper understanding of the biological functions of the DEGs, we performed GO and KEGG pathway enrichment analysis on these genes. Initially, we conducted a Gene Ontology enrichment analysis to explore their roles in BP, CC, and MF. The GO results (Figure 3A, Table S1) show that in BP, DEGs are mainly involved in biological processes such as carbon dioxide transport, oxygen transport, and erythrocyte development. The CC analysis indicates that DEGs are predominantly enriched in the spectrin-associated cytoskeleton and hemoglobin complex. In MF, DEGs are largely enriched in functions like hemoglobin binding, oxygen transporter activity, and ubiquitin-protein transferase activity. Additionally, the KEGG results reveal pathways related to high-altitude acclimatization involving metabolic pathways, mitophagy—animal, and porphyrin metabolism (Figure 3B, Table S2). Among these, the metabolic pathways involve 45 DEGs, making it the pathway with the most differentially expressed genes affected during the acclimatization process to hypoxia.

### 3.3. Significant Differentially Expressed Genes

**Figure 4:**
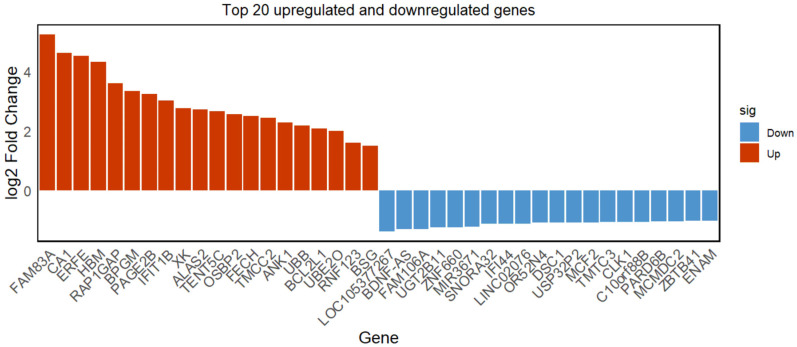
Top 20 significantly upregulated and downregulated genes. Figure 4 shows the top 20 significantly upregulated and downregulated genes, respectively. The top 20 genes with the smallest *p*-values were selected to represent their significance in upregulation and downregulation, which are sorted by log_2_ (FC) values.

Figure 4 displays the top 20 significantly upregulated and downregulated genes, which show significant differences in expression levels compared with the control group. For instance, *CA1*, a carbonic anhydrase, primarily facilitates the regulation between carbon dioxide (CO_2_) and bicarbonate (HCO_3_^−^). Studies have found that inhibiting carbonic anhydrase with acetazolamide can improve ventilation and oxygenation levels, thus reducing oxidative stress [18]. Bisphosphoglycerate mutase (BPGM) is a key rate-limiting enzyme in the Rapoport–Luebering pathway, responsible for producing 2,3-bisphosphoglycerate (2,3-BPG), and 2,3-BPG is a negative allosteric regulator that decreases the affinity of oxygen to hemoglobin (Hb). In hypoxic conditions, the increase in red blood cell O_2_ release is mediated by adaptive hypoxic metabolic reprogramming, which leads to the activation of *BPGM* [19]. Erythroferrone (ERFE), derived from red blood cells, is an important regulator of iron metabolism. Hypoxic exposure can stimulate the adrenal medulla to synthesize and secrete EPO, and an increase in EPO can promote reticulocyte expression and secretion of ERFE [20]. ERFE can inhibit the synthesis of hepcidin, thereby stabilizing the levels of the iron release protein (FPN1), increasing iron absorption and mobilization, meeting the iron demands for hemoglobin synthesis, and promoting erythropoiesis [21]. The upregulation of ERFE during the acclimatization process to high-altitude hypoxia can increase iron levels in the blood, which can be used for heme and hemoglobin synthesis, accelerating erythropoiesis, enhancing the body’s oxygen-carrying capacity, alleviating discomfort caused by high-altitude hypoxia, and promoting the acclimatization process.

### 3.4. Analysis of Protein Interaction Networks in Key Pathways

**Figure 5:**
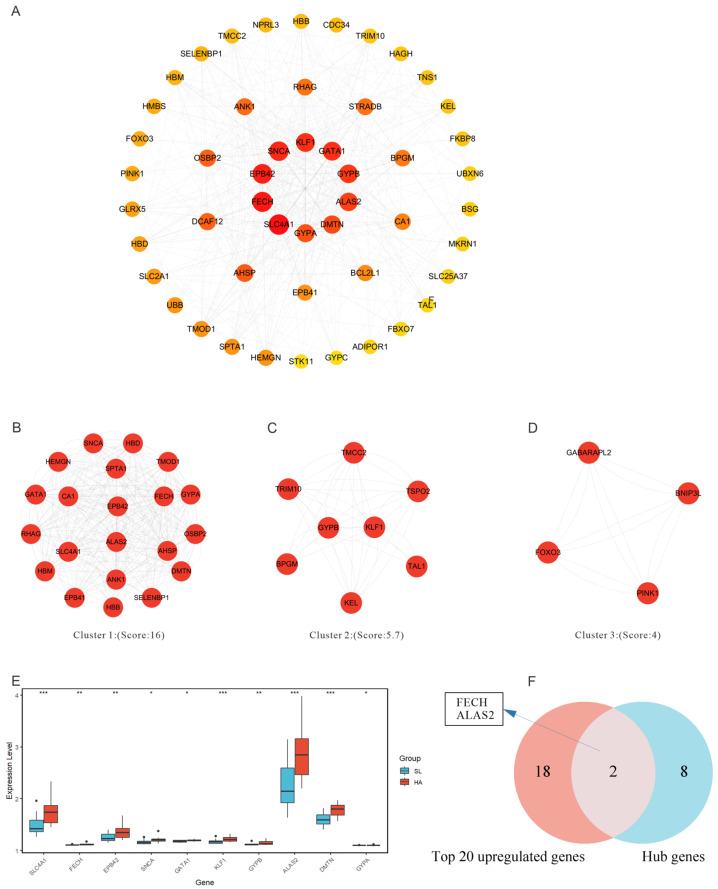
Analysis of protein interaction networks and key molecular expressions. This network diagram is based on the GO and KEGG pathway analysis results under high-altitude conditions. The network analysis results for significant genes display the interaction patterns among significant genes within the cell. The nodes in the diagram represent individual genes, and the connections among nodes represent protein interactions identified through scientific literature and bioinformatics predictions (**A**). Highly interconnected sub-networks identified within the network. Each sub-network’s score indicates the connectivity density and the strength of interactions among the nodes in the network (**B**–**D**). The expression levels of key genes under different conditions (HA and SL) with asterisks indicating statistical significance as follows: *** denotes very significant (*p* < 0.001), ** denotes significant (*p* < 0.01), and * denotes moderately significant (*p* < 0.05) (**E**). Venn diagram illustrating the common genes between key genes and the top 20 DEGs (**F**).

To elucidate the interactions among differentially expressed genes, we constructed a protein interaction network using the STRING database (Figure 5A). Based on the results of the enrichment analysis and calculated degree values, we identified key DEGs within the pathways that exhibited significant changes in the GO and KEGG analysis results (Table 2). Notably, *SLC4A1* emerged as the most critical gene with a degree value of 94. This gene belongs to the Anion Exchanger (AE) family, and its primary function is to regulate the transmembrane transport of anions (such as chloride ions and bicarbonate ions) on the red blood cell membrane, thereby participating in the transport of carbon dioxide from tissues to the lungs. We then focused on highly connected sub-networks that display protein synergistic actions critical for erythrocyte development and heme synthesis (Figure 5B), red blood cell function and structure (Figure 5C), and cell death and survival (Figure 5D). After sorting genes by degree in the network analysis, we determined the expression levels of the top ten key genes, as shown in Figure 5E. The Venn diagram (Figure 5F) illustrates that among the top 20 upregulated genes, two genes, *FECH* and *ALAS2*, overlap with the ten key genes. These genes play a significant role in the heme biosynthetic process pathway, facilitating acclimatization to high altitudes.

### 3.5. Candidate Targets for High-Altitude Hypoxia Acclimatization

**Figure 6:**
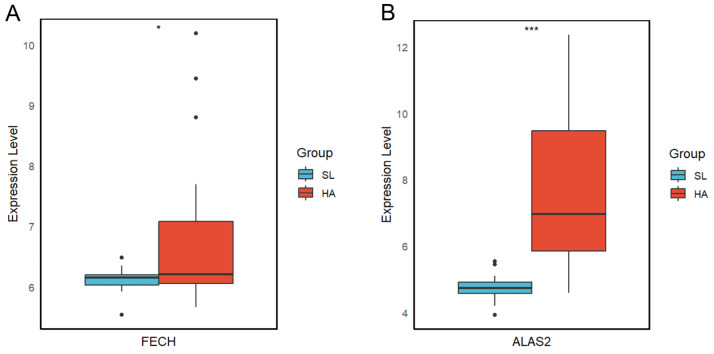
Boxplots of *FECH* and *ALAS2* gene expression levels in plains and at high altitudes. Boxplot showing the expression level of the *FECH* gene in plains (SL) and high-altitude (HA) environments. The expression levels are generally higher in the HA group, indicated by a single asterisk (*), representing a *p*-value < 0.05 (**A**). Boxplot displaying the expression level of the *ALAS2* gene in the same environments. There is a significant increase in expression levels in the HA group, as indicated by the asterisks (***), representing a *p*-value < 0.001 (**B**).

In order to validate the two key genes screened, we analyzed the expression of these two genes in the GSE103927 dataset. The GSE103927 dataset was processed to extract the expression levels of the *FECH* and *ALAS2* genes, both of which showed upregulation with *p* < 0.05 (Figure 6).

## 4. Discussion

Upon ascending from plains to plateau regions, the reduction in atmospheric pressure leads to a decrease in the partial pressure of oxygen in the air [22], resulting in less oxygen being inhaled during respiration and causing hypoxia at the tissue and cellular levels [23]. In response to the hypoxic environment of the plateau, the human body demonstrates a strong adaptive capacity. To intake and transport more oxygen, physiological responses, such as increased respiration and heart rate, and an increase in red blood cells occur [24,25]. Although progress has been made in studies on acclimatization to high altitudes, the systematic changes in gene expression levels and key pathways during rapid acclimatization to hypoxic environments are still unclear. This study focuses on the changes in gene expression as people from low-altitude areas ascend to high-altitude environments. Initially, three datasets meeting the criteria were selected from public databases, and bioinformatics methods were used to identify genes potentially related to acclimatization to hypoxic plateau environments. Our analysis identified 361 upregulated and 21 downregulated DEGs, shedding light on the molecular mechanisms underpinning adaptation to high altitudes.

In the KEGG analysis results, it was discovered that the mitophagy—animal pathway is significantly upregulated. Studies have shown that the activation of mitophagy can ultimately inhibit apoptosis, thus promoting pulmonary vascular remodeling (PVR) [26,27]. Specifically, PTEN-induced kinase 1 (*PINK1*) is observed aggregating on the mitochondrial membrane, which leads to the recruitment of cytoplasmic Parkin into the mitochondria, followed by the ubiquitination of damaged mitochondria [28]. In our findings, PINK1 showed significant upregulation, suggesting that the enhanced activation of the mitophagy—animal pathway may play a pivotal role in facilitating acclimatization at high altitudes. Moreover, the pathway for glycine, serine, and threonine metabolism was also significantly upregulated. The overactivation of serine/glycine biosynthesis provides single-carbon units for one-carbon metabolism, where serine contributes to porphyrin synthesis [29]. One-carbon metabolism is a complex cyclic metabolic network based on the chemical reaction of folate compounds, supplying essential proteins, nucleic acids, lipids, and other biomacromolecules to the organism [30]. The upregulation of this pathway may enhance porphyrin production and maintain redox homeostasis. The KEGG results emphasize enhancements in cellular homeostasis and metabolic processes, critical for adapting to high-altitude hypoxia.

The results of the GO analysis revealed significantly upregulated pathways like carbon dioxide transport and oxygen transport. In carbon dioxide transport, carbonic anhydrase (*CA1*) plays a role in regulating the concentrations of CO_2_, HCO_3_^−^, and hydrogen ions (H^+^) inside and outside cells, thus adjusting the acid–base balance and pH levels [31]. Various acid–base balance membrane proteins, including the SLC4 family transporter *SLC4A1*, are regulated by carbonic anhydrase [32]. As blood is transported from tissues to the lungs, carbon dioxide enters the red blood cells and is catalyzed by carbonic anhydrase into carbonic acid, which quickly decomposes into water and bicarbonate. The bicarbonate is then exchanged into the plasma through *SLC4A1*, and chloride ions move into the red blood cells [33,34], supporting efficient carbon dioxide transport and release and ensuring effective clearance of carbon dioxide from tissues and its expulsion from the lungs at high altitudes. The oxygen transport pathway ranked highly in GO fold enrichment, indicating that the body might upregulate oxygen transport-related genes to adapt to the stress of high-altitude environments. Oxygen transport primarily occurs through the binding of oxygen to hemoglobin. In high-altitude environments, hemoglobin interacts with 2,3-BPG to adapt to low-oxygen conditions [31]. Low-oxygen conditions stimulate the kidneys to produce more EPO, thus promoting the production of red blood cells and thereby increasing the production of 2,3-BPG. Increased 2,3-BPG reduces hemoglobin’s affinity for oxygen [35], enhancing the body’s capability to utilize the available oxygen more effectively and mitigating symptoms of altitude sickness. In summary, the substantial upregulation of both carbon dioxide and oxygen transport pathways highlights a critical adaptive mechanism, optimizing gas exchange and maintaining physiological homeostasis in response to the reduced oxygen availability at high altitudes. This adaptation ensures efficient cellular function and survival under hypoxic conditions.

A significant portion of the biological pathways enriched in this study are involved in the development and differentiation of red blood cells. In mammals, the differentiation of hematopoietic stem progenitor cells into the erythroid lineage involves the process of enucleation from nucleated red blood cells to mature ones [36]. The primary function of red blood cells is oxygen transport; the ferrous ion in heme can reversibly bind oxygen molecules, forming oxyhemoglobin and deoxyhemoglobin, thereby facilitating oxygen transport and release. However, certain stressors can significantly impact this crucial functionality. For instance, some stressors lead to a marked decrease in the intensity of the hemoglobin monomer, which is associated with the formation of its tetrameric form [37]. This structural alteration could potentially have detrimental effects on erythrocyte physiology, primarily by altering the oxygen-binding capacity of hemoglobin. Central to red blood cell development is the synthesis of heme. *GATA1* and *KLF1* are key transcription factors [38], and *KLF1* enhances β-globin gene expression, complementing *GATA1*′s regulatory effects, which collectively ensure efficient hemoglobin synthesis [39,40]. This synergistic action ensures the efficient synthesis of hemoglobin. In addition, 5-Aminolevulinate synthase (ALAS) 2 is the rate-limiting enzyme in the biosynthesis of heme, catalyzing the production of the heme precursor 5-aminolevulinate (5-ALA) [41]. Variability in *ALAS2*, as demonstrated in a study of 244 participants, correlates with differential responses to HiHiLo training [42]. It can be speculated that *ALAS2* may affect hemoglobin levels and promote high-altitude acclimatization. On the one hand, *GATA1* can activate the transcription of *ALAS2*, promoting heme production and mediating red cell maturation [43]; on the other hand, researchers have found that high levels of heme can suppress the expression of the *GATA1* gene and mitotic spindle genes, thereby inhibiting erythropoiesis [44]. *GATA1* and heme have a direct feedback regulation loop. *ALAS2* then catalyzes the formation of aminolevulinic acid (ALA), the first step in heme synthesis. In the final stage of heme synthesis, ferrochelatase (*FECH*) catalyzes the insertion of ferrous ions into protoporphyrin IX in the mitochondria of red blood cells to form heme. For instance, the interaction between *FECH* and Abcb7 is critical for regulating iron distribution within cells, facilitating iron–sulfur cluster metabolism, which is essential for cellular energy processes [45,46]. Furthermore, there is a synergistic interaction between *FECH* and *ALAS2* [47,48]. Studies consistently show that exposure to high altitudes results in increased red blood cell counts and hemoglobin levels [49,50]. Following intermittent hypoxic exposure, the hemoglobin concentration also tends to increase and shows a significant correlation with exposure time [51]. The upregulation of *GATA1*, *ALAS2*, and *FECH* during acclimatization to high altitudes may promote heme production and facilitate the development and differentiation of red blood cells, thereby aiding the body’s adaptation to high altitudes.

However, free heme is toxic and can cause an increase in reactive oxygen species (ROS), leading to oxidative stress damage as well as apoptosis and ferroptosis. Heme synthesis must be closely coordinated with globin synthesis. *FLVCR1* is a heme export protein that can transport free heme out of the cell while retaining the function of heme as a signaling molecule and protein cofactor [52]. In this study, it was found that the expression of *FLVCR1* was downregulated in the HA group, and the heme synthesis pathway was upregulated. We speculate that part of the oxidative stress damage and apoptosis observed under hypoxia is mediated by the elevation of intracellular free heme.

## 5. Conclusions

This study analyzed three datasets from the GEO database to uncover significant pathways and key genes enhancing human acclimatization to high altitudes. We identified critical pathways involved in blood gas transport, erythrocyte development and differentiation, and the heme biosynthetic process. Notably, *FECH* and *ALAS2*, which are central to the heme biosynthetic process, were not only among the top 20 upregulated genes but featured prominently among the ten key genes identified in the network analysis. This dual presence underscores their critical role in the acclimatization process at high altitudes, illustrating their significant impact on the body’s adaptation mechanisms. The overlap of these genes in both categories highlights their pivotal functions and suggests that the heme biosynthetic process is essential for adapting to hypoxic conditions. These findings provide new biological insights into the mechanisms of human adaptation to high altitudes and suggest potential targets for enhancing acclimatization, contributing valuable information for future research and practical applications in acclimatization strategies.

## 6. Limitations

This study on acute high-altitude exposure has certain limitations that must be acknowledged. Firstly, the dataset used in this research was limited to data collected on the third day after arrival at high altitudes. While this timeframe provides valuable insights into the early stages of acclimatization, it does not capture the complete temporal dynamics of physiological adaptation. Future studies should consider extending the observation period to include additional time points, such as seven or twenty-one days, to better understand the progression of acclimatization over time. Secondly, the altitude range of the dataset spanned from 3000 to 5300 m. Although this range covers moderate to high altitudes, further stratification into more specific altitude categories could provide more detailed insights into altitude-specific physiological responses.

## Figures and Tables

**Table 1:** Basic characteristics of all participants.

Dataset	Age *	n	Height *	Weight *	SBP *	DBP *	HR *	SpO_2_ *	Smoker/Drinker
GSE133702	19–23	30	—	—	—	—	—	—	No
GSE103940	21.6 ± 2.0	53	172 ± 1.0	64.9 ± 2.3	—	—	—	—	—
GSE75665	20–23	10	—	—	116 ± 15.0	58 ± 5.7	65 ± 10.1	97 ± 1.0	—
total number of participants	—	93	—	—	—	—	—	—	

* Age: years. Height: cm. Weight: kg. Systolic blood pressure (SBP): mmHg. Diastolic blood pressure (DBP): mmHg. Heart rate (HR): bpm. pulse oxygen saturation (SpO_2_): %. Values are shown as the mean ± standard deviation.

**Table 2:** Information about key genes.

Gene Name	Description	Expression Level *	Degree
*SLC4A1*	solute carrier family 4 member 1 (Diego blood group)	Up	94
*FECH*	ferrochelatase	Up	90
*EPB42*	erythrocyte membrane protein band 4.2	Up	86
*SNCA*	synuclein α	Up	84
*GATA1*	GATA binding protein 1	Up	76
*KLF1*	KLF transcription factor 1	Up	76
*GYPB*	glycophorin B (MNS blood group)	Up	74
*ALAS2*	5′-aminolevulinate synthase 2	Up	72
*DMTN*	dematin actin binding protein	Up	70
*GYPA*	glycophorin A (MNS blood group)	Up	68

* Expression level: the term “Up” represents “Upregulated”, which indicates a significant increase in the expression level of specific genes when compared with their levels in the control group at lower altitudes.

## Data Availability

The datasets described in this article are available from Gene Expression Omnibus (https://www.ncbi.nlm.nih.gov/geo/, accessed on 11 November 2023) via the accession numbers GSE133702, GSE103940, GSE75665, and GSE103927. All data generated in this article are freely accessible in supplementary tables to any scientist wishing to use them noncommercially. On reasonable request, the corresponding author can provide further information.

## Supplementary Materials

The following supporting information can be downloaded at https://www.mdpi.com/article/10.3390/genes15081075/s1, Figure S1: Integration of datasets to remove batch effects; Table S1: GO pathway analysis results; Table S2: KEGG pathway analysis results.

## References

[1] Bärtsch P., Swenson E.R. (2013). Clinical practice: Acute high-altitude illnesses. N. Engl. J. Med..

[2] Savioli G., Ceresa I.F., Gori G., Fumoso F., Gri N., Floris V., Varesi A., Martuscelli E., Marchisio S., Longhitano Y. (2022). Pathophysiology and Therapy of High-Altitude Sickness: Practical Approach in Emergency and Critical Care. J. Clin. Med..

[3] Luks A.M., Swenson E.R., Bärtsch P. (2017). Acute high-altitude sickness. Eur. Respir. Rev..

[4] San T., Polat S., Cingi C., Eskiizmir G., Oghan F., Cakir B. (2013). Effects of high altitude on sleep and respiratory system and theirs adaptations. Sci. World J..

[5] Naeije R. (2010). Physiological adaptation of the cardiovascular system to high altitude. Prog. Cardiovasc. Dis..

[6] Richalet J.P., Hermand E., Lhuissier F.J. (2024). Cardiovascular physiology and pathophysiology at high altitude. Nat. Rev. Cardiol..

[7] Gassmann M., Mairbäurl H., Livshits L., Seide S., Hackbusch M., Malczyk M., Kraut S., Gassmann N.N., Weissmann N., Muckenthaler M.U. (2019). The increase in hemoglobin concentration with altitude varies among human populations. Ann. N. Y. Acad. Sci..

[8] Zhao Y., Wang X., Noviana M., Hou M. (2018). Nitric oxide in red blood cell adaptation to hypoxia. Acta Biochim. Biophys. Sin..

[9] Masson N., Willam C., Maxwell P.H., Pugh C.W., Ratcliffe P.J. (2001). Independent function of two destruction domains in hypoxia-inducible factor-alpha chains activated by prolyl hydroxylation. EMBO J..

[10] Azad P., Caldwell A.B., Ramachandran S., Spann N.J., Akbari A., Villafuerte F.C., Bermudez D., Zhao H., Poulsen O., Zhou D. (2022). ARID1B, a molecular suppressor of erythropoiesis, is essential for the prevention of Monge’s disease. Exp. Mol. Med..

[11] Weidemann A., Johnson R.S. (2008). Biology of HIF-1alpha. Cell Death Differ..

[12] Liu F., Hu C., Ding J., Fu C., Wang S., Li T. (2023). GATA-1 Promotes Erythroid Differentiation Through the Upregulation of miR-451a and miR-210-3p Expressions in CD34(+) Cells in High-Altitude Polycythemia. High. Alt. Med. Biol..

[13] Taylor C.T. (2008). Mitochondria and cellular oxygen sensing in the HIF pathway. Biochem. J..

[14] Gaur P., Saini S., Ray K., Asanbekovna K.N., Akunov A., Maripov A., Sarybaev A., Singh S.B., Kumar B., Vats P. (2020). Temporal transcriptome analysis suggest modulation of multiple pathways and gene network involved in cell-cell interaction during early phase of high altitude exposure. PLoS ONE.

[15] Yasukochi Y., Shin S., Wakabayashi H., Maeda T. (2020). Transcriptomic Changes in Young Japanese Males After Exposure to Acute Hypobaric Hypoxia. Front. Genet..

[16] Burtscher J., Hohenauer E., Burtscher M., Millet G.P., Egg M. (2023). Environmental and behavioral regulation of HIF-mitochondria crosstalk. Free Radic. Biol. Med..

[17] Sharma V., Varshney R., Sethy N.K. (2022). Human adaptation to high altitude: A review of convergence between genomic and proteomic signatures. Hum. Genom..

[18] Lu H., Zhang H., Jiang Y. (2020). Methazolamide in high-altitude illnesses. Eur. J. Pharm. Sci..

[19] Xu P., Chen C., Zhang Y., Dzieciatkowska M., Brown B.C., Zhang W., Xie T., Abdulmalik O., Song A., Tong C. (2022). Erythrocyte transglutaminase-2 combats hypoxia and chronic kidney disease by promoting oxygen delivery and carnitine homeostasis. Cell Metab..

[20] Kautz L., Jung G., Valore E.V., Rivella S., Nemeth E., Ganz T. (2014). Identification of erythroferrone as an erythroid regulator of iron metabolism. Nat. Genet..

[21] Kautz L., Jung G., Du X., Gabayan V., Chapman J., Nasoff M., Nemeth E., Ganz T. (2015). Erythroferrone contributes to hepcidin suppression and iron overload in a mouse model of β-thalassemia. Blood.

[22] Peacock A.J. (1998). ABC of oxygen: Oxygen at high altitude. BMJ.

[23] Sarkar M., Niranjan N., Banyal P.K. (2017). Mechanisms of hypoxemia. Lung India.

[24] Zhang Z.A., Sun Y., Yuan Z., Wang L., Dong Q., Zhou Y., Zheng G., Aschner M., Zou Y., Luo W. (2022). Insight into the Effects of High-Altitude Hypoxic Exposure on Learning and Memory. Oxid. Med. Cell Longev..

[25] West J.B. (2012). High-altitude medicine. Am. J. Respir. Crit. Care Med..

[26] Linqing L., Yuhan Q., Erfei L., Yong Q., Dong W., Chengchun T., Gaoliang Y., Bo L. (2021). Hypoxia-induced PINK1/Parkin-mediated mitophagy promotes pulmonary vascular remodeling. Biochem. Biophys. Res. Commun..

[27] Saraji A., Sydykov A., Schäfer K., Garcia-Castro C.F., Henneke I., Alebrahimdehkordi N., Kosanovic D., Hadzic S., Guenther A., Hecker M. (2021). PINK1-mediated Mitophagy Contributes to Pulmonary Vascular Remodeling in Pulmonary Hypertension. Am. J. Respir. Cell Mol. Biol..

[28] Greene A.W., Grenier K., Aguileta M.A., Muise S., Farazifard R., Haque M.E., McBride H.M., Park D.S., Fon E.A. (2012). Mitochondrial processing peptidase regulates PINK1 processing, import and Parkin recruitment. EMBO Rep..

[29] Zhang Y., Yu H., Zhang J., Gao H., Wang S., Li S., Wei P., Liang J., Yu G., Wang X. (2021). Cul4A-DDB1-mediated monoubiquitination of phosphoglycerate dehydrogenase promotes colorectal cancer metastasis via increased S-adenosylmethionine. J. Clin. Investig..

[30] Pan S., Fan M., Liu Z., Li X., Wang H. (2021). Serine, glycine and one-carbon metabolism in cancer (Review). Int. J. Oncol..

[31] Kimura H., Hamasaki N., Yamamoto M., Tomonaga M. (1995). Circulation of red blood cells having high levels of 2,3-bisphosphoglycerate protects rat brain from ischemic metabolic changes during hemodilution. Stroke.

[32] Becker H.M., Klier M., Deitmer J.W. (2014). Carbonic anhydrases and their interplay with acid/base-coupled membrane transporters. Subcell. Biochem..

[33] Seifter J.L., Chang H.Y. (2017). Extracellular Acid-Base Balance and Ion Transport Between Body Fluid Compartments. Physiology.

[34] Kalli A.C., Reithmeier R.A.F. (2018). Interaction of the human erythrocyte Band 3 anion exchanger 1 (AE1, SLC4A1) with lipids and glycophorin A: Molecular organization of the Wright (Wr) blood group antigen. PLoS Comput. Biol..

[35] Faggiano S., Ronda L., Bruno S., Abbruzzetti S., Viappiani C., Bettati S., Mozzarelli A. (2022). From hemoglobin allostery to hemoglobin-based oxygen carriers. Mol. Aspects Med..

[36] Dzierzak E., Philipsen S. (2013). Erythropoiesis: Development and differentiation. Cold Spring Harb. Perspect. Med..

[37] Piscopo M., Notariale R., Tortora F., Lettieri G., Palumbo G., Manna C. (2020). Novel Insights into Mercury Effects on Hemoglobin and Membrane Proteins in Human Erythrocytes. Molecules.

[38] Ludwig L.S., Lareau C.A., Bao E.L., Nandakumar S.K., Muus C., Ulirsch J.C., Chowdhary K., Buenrostro J.D., Mohandas N., An X. (2019). Transcriptional States and Chromatin Accessibility Underlying Human Erythropoiesis. Cell Rep..

[39] Vinjamur D.S., Alhashem Y.N., Mohamad S.F., Amin P., Williams D.C., Lloyd J.A. (2016). Krüppel-Like Transcription Factor KLF1 Is Required for Optimal γ- and β-Globin Expression in Human Fetal Erythroblasts. PLoS ONE.

[40] Gnanapragasam M.N., Planutis A., Glassberg J.A., Bieker J.J. (2023). Identification of a genomic DNA sequence that quantitatively modulates KLF1 transcription factor expression in differentiating human hematopoietic cells. Sci. Rep..

[41] Stojanovski B.M., Hunter G.A., Na I., Uversky V.N., Jiang R.H.Y., Ferreira G.C. (2019). 5-Aminolevulinate synthase catalysis: The catcher in heme biosynthesis. Mol. Genet. Metab..

[42] Xu Y., Hu Y., Ren Z., Yi L. (2015). Delta-aminolevulinate synthase 2 polymorphism is associated with maximal oxygen uptake after Living-high exercise-high training-low in a male Chinese population. Int. J. Clin. Exp. Med..

[43] Tanimura N., Miller E., Igarashi K., Yang D., Burstyn J.N., Dewey C.N., Bresnick E.H. (2016). Mechanism governing heme synthesis reveals a GATA factor/heme circuit that controls differentiation. EMBO Rep..

[44] Doty R.T., Yan X., Lausted C., Munday A.D., Yang Z., Yi D., Jabbari N., Liu L., Keel S.B., Tian Q. (2019). Single-cell analyses demonstrate that a heme-GATA1 feedback loop regulates red cell differentiation. Blood.

[45] Obi C.D., Bhuiyan T., Dailey H.A., Medlock A.E. (2022). Ferrochelatase: Mapping the Intersection of Iron and Porphyrin Metabolism in the Mitochondria. Front. Cell Dev. Biol..

[46] Medlock A.E., Shiferaw M.T., Marcero J.R., Vashisht A.A., Wohlschlegel J.A., Phillips J.D., Dailey H.A. (2015). Identification of the Mitochondrial Heme Metabolism Complex. PLoS ONE.

[47] Medlock A.E., Dailey H.A. (2022). New Avenues of Heme Synthesis Regulation. Int. J. Mol. Sci..

[48] Obi C.D., Dailey H.A., Jami-Alahmadi Y., Wohlschlegel J.A., Medlock A.E. (2023). Proteomic Analysis of Ferrochelatase Interactome in Erythroid and Non-Erythroid Cells. Life.

[49] Saunders P.U., Garvican-Lewis L.A., Schmidt W.F., Gore C.J. (2013). Relationship between changes in haemoglobin mass and maximal oxygen uptake after hypoxic exposure. Br. J. Sports Med..

[50] Siqués P., Brito J., León-Velarde F., Barrios L., De La Cruz J.J., López V., Herruzo R. (2007). Hematological and lipid profile changes in sea-level natives after exposure to 3550-m altitude for 8 months. High Alt. Med. Biol..

[51] Akunov A., Sydykov A., Toktash T., Doolotova A., Sarybaev A. (2018). Hemoglobin Changes After Long-Term Intermittent Work at High Altitude. Front. Physiol..

[52] Keel S.B., Doty R.T., Yang Z., Quigley J.G., Chen J., Knoblaugh S., Kingsley P.D., De Domenico I., Vaughn M.B., Kaplan J. (2008). A heme export protein is required for red blood cell differentiation and iron homeostasis. Science.

