# Supplementary material for: Key Genes FECH and ALAS2 under Acute High-Altitude Exposure: A Gene Expression and Network Analysis Based on Expression Profile Data
Source: Genes (Basel). 2024 Aug 14;15(8):1075. doi: 10.3390/genes15081075 (PMC11353374; doi:10.3390/genes15081075)
Supplement: Supplementary file 1 [file genes-15-01075-s001.zip › Table S1 S2.pdf]

**Table S1.** GO Pathway Analysis Results.

| Pathway name | Count | PValue | Fold Enrichment | FDR | GO |
|---|---|---|---|---|---|
| carbon dioxide transport | 6 | 2.38E-06 | 25.24 | 3.34E-03 | BP |
| oxygen transport | 5 | 1.25E-04 | 18.40 | 3.69E-02 | BP |
| erythrocyte development | 6 | 2.20E-05 | 16.82 | 1.30E-02 | BP |
| heme biosynthetic process | 6 | 5.42E-05 | 14.13 | 2.40E-02 | BP |
| hydrogen peroxide catabolic process | 6 | 9.62E-05 | 12.62 | 3.41E-02 | BP |
| erythrocyte differentiation | 9 | 3.77E-06 | 9.64 | 3.34E-03 | BP |
| positive regulation of ATPase activity | 4 | 8.39E-03 | 9.42 | 6.75E-01 | BP |
| negative regulation of cysteine-type endopeptidase activity involved in apoptotic process | 6 | 6.86E-04 | 8.41 | 1.41E-01 | BP |
| mitophagy | 5 | 6.43E-03 | 6.69 | 6.06E-01 | BP |
| ubiquitin-dependent protein catabolic | 13 | 6.25E-04 | 3.30 | 1.41E-01 | BP |
| spectrin-associated cytoskeleton | 5 | 5.27E-06 | 36.96 | 5.73E-04 | CC |
| hemoglobin complex | 5 | 6.96E-05 | 21.12 | 4.54E-03 | CC |
| cortical cytoskeleton | 7 | 9.64E-07 | 19.71 | 1.57E-04 | CC |
| haptoglobin-hemoglobin complex | 4 | 1.20E-03 | 18.20 | 4.34E-02 | CC |
| mitochondrial outer membrane | 14 | 9.40E-05 | 3.78 | 5.11E-03 | CC |
| blood microparticle | 9 | 3.54E-03 | 3.62 | 1.02E-01 | CC |
| mitochondrion | 38 | 6.08E-03 | 1.57 | 1.32E-01 | CC |
| cytosol | 138 | 1.90E-07 | 1.47 | 6.18E-05 | CC |
| cytoplasm | 130 | 3.86E-05 | 1.36 | 3.15E-03 | CC |
| membrane | 124 | 4.08E-04 | 1.31 | 1.90E-02 | CC |
| hemoglobin binding | 3 | 3.01E-03 | 33.82 | 1.76E-01 | MF |
| organic acid binding | 4 | 8.14E-04 | 20.50 | 1.21E-01 | MF |
| oxygen transporter activity | 4 | 2.59E-03 | 14.09 | 1.76E-01 | MF |
| spectrin binding | 5 | 1.06E-03 | 10.84 | 1.21E-01 | MF |
| amino acid transmembrane transporter | 5 | 2.62E-03 | 8.54 | 1.76E-01 | MF |
| structural constituent of cytoskeleton | 10 | 1.47E-04 | 5.12 | 4.22E-02 | MF |
| protein dimerization activity | 11 | 1.73E-03 | 3.35 | 1.65E-01 | MF |
| ubiquitin-protein transferase activity | 13 | 9.83E-04 | 3.13 | 1.21E-01 | MF |
| actin binding | 15 | 3.08E-03 | 2.49 | 1.76E-01 | MF |
| protein binding | 264 | 2.12E-06 | 1.17 | 1.21E-03 | MF |

**Table S2.** KEGG Pathway Analysis Results.

| Pathway name | Count | PValue | Fold Enrichment | FDR |
|---|---|---|---|---|
| Metabolic pathways | 45 | 4.70E-03 | 1.48 | 3.01E-01 |
| Biosynthesis of cofactors | 9 | 1.01E-02 | 3.00 | 3.68E-01 |
| Mitophagy-animal | 8 | 3.92E-03 | 3.96 | 3.01E-01 |

| | | | | |
|---|---|---|---|---|
| Bile secretion | 7 | 7.72E-03 | 4.01 | 3.30E-01 |
| Ubiquitin mediated proteolysis | 7 | 5.91E-02 | 2.51 | 9.96E-01 |
| Porphyrin metabolism | 6 | 1.41E-03 | 7.11 | 3.01E-01 |
| Glycine, serine and threonine metabolism | 5 | 7.40E-03 | 6.37 | 3.30E-01 |
| Arginine and proline metabolism | 5 | 1.60E-02 | 5.10 | 5.13E-01 |
| Adipocytokine signaling pathway | 5 | 4.75E-02 | 3.64 | 9.96E-01 |
| Biosynthesis of nucleotide sugars | 4 | 3.49E-02 | 5.51 | 9.93E-01 |
