# Supplementary figures and images for: Key Genes FECH and ALAS2 under Acute High-Altitude Exposure: A Gene Expression and Network Analysis Based on Expression Profile Data
Source: Genes (Basel). 2024 Aug 14;15(8):1075. doi: 10.3390/genes15081075 (PMC11353374; doi:10.3390/genes15081075)

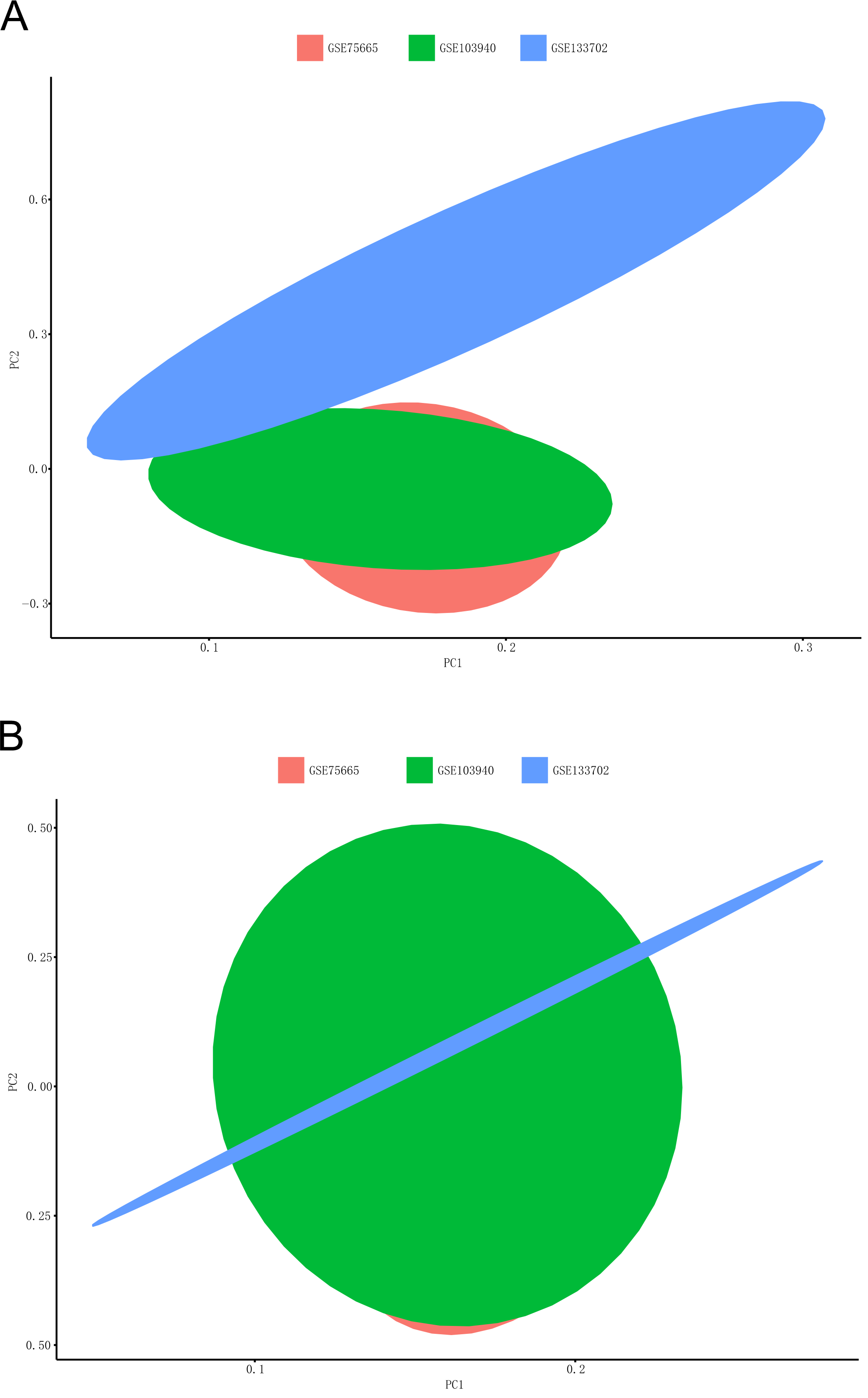

Supplement: Supplementary file 1 [file genes-15-01075-s001.zip › Figure S1.tif]
